# Optimizing Essential Oil Mixtures: Synergistic Effects on Cattle Rumen Fermentation and Methane Emission

**DOI:** 10.3390/ani15142105

**Published:** 2025-07-16

**Authors:** Memoona Nasir, María Rodríguez-Prado, Marica Simoni, Susana M. Martín-Orúe, José Francisco Pérez, Sergio Calsamiglia

**Affiliations:** 1 Animal Nutrition and Welfare Service (SNiBA), Department of Animal and Food Science, Universitat Autònoma de Barcelona (UAB), 08193 Bellaterra, Spain; maria.rodriguez.prado@uab.cat (M.R.-P.); susana.martin@uab.cat (S.M.M.-O.); josefrancisco.perez@uab.cat (J.F.P.); 2 Department of Veterinary Science, University of Parma, 46126 Parma, Italy; marica.simoni@unipr.it

**Keywords:** essential oils, feed additives, methane mitigation, rumen fermentation, synergistic effects

## Abstract

Ruminant livestock contribute significantly to methane emissions, necessitating sustainable mitigation strategies. Essential oils (EOs) show promise for modulating ruminal fermentation, but their synergistic effects remain underexplored. Two 24 h in vitro experiments evaluated the synergistic effects of EO blends on rumen microbial fermentation. Exp. 1 screened five oils using two triad combinations. Triad 1 tested 10 combinations of thyme (THY), peppermint (PPM), and cinnamon leaf (CIN) oils. Triad 2 tested 10 combinations of anise (ANI), clove leaf (CLO), and peppermint (PPM) oils. Each blend was tested at 400 mg/L, using batch culture methods measuring: pH, ammonia-N (NH_3_-N), and volatile fatty acids (VFAs). The two most effective blends, designated as T1 and T2, were selected for Exp. 2 to assess total gas and methane (CH_4_) production using pressure transducer methods. All treatments were incubated in a rumen fluid–buffer mix with a 50:50 forage-to-concentrate substrate (pH 6.6). In Exp. 1, data were analyzed according to the Simplex Centroid Design using R-Studio. In Exp. 2, an analysis was conducted using the MIXED procedure in SAS. Mean comparisons were assessed through Tukey’s test. The results from Exp. 1 identified CIN+PPM (80:20) and ANI+CLO (80:20) as optimal combinations, both increasing total VFAs while reducing acetate/propionate ratios and NH_3_-N concentrations. In Exp. 2, both combinations significantly reduced total gas and CH_4_ productions compared to the control, with CIN+PPM achieving the greatest methane reduction (similar to monensin, the positive control). Specific essential oil combinations demonstrated synergistic effects in modulating rumen fermentation and reducing methane emissions, offering potential for sustainable livestock production. Further in vivo validation is required to optimize dosing and assess long-term effects on animal performance.

## Simple Summary

Unlocking the potential of essential oil combinations in ruminant nutrition presents a transformative approach to optimizing fermentation efficiency while addressing the critical challenge of methane emissions. These natural compounds, when strategically combined, hold promise for reshaping sustainable livestock management by enhancing nutrient utilization and reducing environmental impact. This study explored the synergistic effects of various essential oil mixtures on ruminal fermentation profiles using a systems-level approach to evaluate interacting factors rather than focusing solely on individual parameters. Specific combinations were screened and found to significantly enhance the production of volatile fatty acids, vital for rumen function, while achieving notable reductions in methane emissions. Some essential oils contributed minimally, indicating that not all oils are equally effective in such applications, and careful selection of compatible combinations is essential. Two optimal blends were identified: cinnamon and peppermint oils (80:20) and anise and clove oils (80:20). Both combinations significantly reduced total gas and methane production compared to controls, with the cinnamon–peppermint blend achieving the greatest methane reduction. These results demonstrate the potential of optimized essential oil blends as effective, sustainable alternatives to conventional additives in livestock production.

## 1. Introduction

Ruminant livestock are essential to global agricultural systems, yet they are significant contributors to greenhouse gas (GHG) emissions, particularly methane (CH_4_), which is a potent driver of climate change. Methane emissions from ruminants primarily result from enteric fermentation in the rumen, where methanogenic archaea convert hydrogen and carbon dioxide into CH_4_ during the fermentation of ingested feed [1,2]. This microbial fermentation process is influenced by numerous factors, including diet composition, feed intake, and nutrient utilization efficiency [3,4]. Methane production contributes to environmental issues [5] and represents an energy loss of 2–12% of gross energy intake [6,7,8], thereby reducing animal performance [9,10]. Therefore, it is imperative to focus efforts on enhancing nutrient utilization and mitigating CH_4_ emissions from ruminants by addressing fermentation processes and methanogen activity to achieve sustainability in agriculture and meet global climate goals [11,12,13]. Modifying ruminal microbial fermentation with feed additives represents a potential strategy to enhance energy and protein utilization efficiency [14,15]. Antibiotics and ionophores have been effective in this context, but their social acceptance is low due to their toxicity and development of antimicrobial resistance; their use as growth promoters has been prohibited in the European Union since 2006 [16]. Therefore, there has been increasing interest in safer, more sustainable natural alternatives, such as tannins, saponins, algae-based products, probiotics, zeolites, and essential oils, all of which have shown promising effects on rumen function and animal performance [17,18,19,20]. Furthermore, optimized rumen function has been associated with systemic physiological benefits, including enhanced immune competence, improved reproductive performance, and better overall animal welfare, as reported in the recent literature [21,22,23].

Essential oils (EO) are intricate combinations of low molecular weight volatile organic compounds, synthesized by various plant components [24]. Multiple studies have indicated that EOs can regulate rumen microbial fermentation by reducing acetate, ammonia-N (NH_3_-N), and CH_4_ while increasing propionate and butyrate concentrations [25,26]. This phenomenon is attributed to the potent antimicrobial properties of EOs against a broad spectrum of Gram-positive and Gram-negative bacteria [27]. Moreover, EOs have demonstrated the capacity to impede methanogenesis by disrupting the metabolic activities of methanogens [28,29]. When utilized individually, numerous EOs positively influence rumen fermentation. Notably, three phenylpropanoids (cinnamaldehyde in cinnamon oil, eugenol in clove bud oil, and anethol in anise oil) have exhibited promising effects in altering rumen microbial activities [30]. These findings align with the review by Cobellis et al. [31], which also highlights the potential of phenolic terpenes, such as carvacrol (in oregano oil) and thymol (in thyme oil), to modulate rumen fermentation.

Essential oils (EOs) have been widely studied for their effects on ruminal fermentation using both in vivo and in vitro approaches. In vivo trials offer physiological relevance, enabling direct observation of systemic responses, but they are often resource-intensive [32,33]. In contrast, in vitro methods, particularly batch culture systems, provide a cost-effective, reproducible platform for assessing fermentation processes and microbial ecology under controlled conditions [34,35,36]. These systems simulate key rumen functions and allow precise manipulation of experimental variables such as substrate composition, additive type, and incubation duration [37,38]. Batch culture systems are especially valuable for screening phytogenic feed additives, such as EOs, as they enable direct quantification of fermentation end-products, including total gas, methane (CH_4_), volatile fatty acids (VFAs), and ammonia-N, while also providing insight into underlying microbial processes [39,40]. Accordingly, the present study employed an in vitro batch culture approach to investigate the effects of combined EO-based treatments on key rumen fermentation parameters.

While individual EOs show promise, their combined use may offer enhanced benefits through synergistic interactions. However, there is limited research on their collective use and potential synergies. EOs exhibit diverse effects and mechanisms of action. Calsamiglia et al. [30] proposed that a cautious combination may result in synergistic effects that enhance ruminal fermentation. For instance, the combination of thymol and cinnamaldehyde demonstrates synergy by facilitating each other’s action: thymol or carvacrol could increase the permeability of the cytoplasmic membrane, potentially aiding the transport of cinnamaldehyde into the cell [41]. Furthermore, when combined, menthol in peppermint oil amplifies the antimicrobial action of thymol and cinnamaldehyde by disrupting microbial cell membranes [42].

We hypothesized that combining specific EOs, namely, thyme oil, cinnamon leaf oil, clove leaf oil, anise oil, and peppermint oil, would produce synergistic effects, enhancing nutrient utilization while reducing CH_4_ emissions. This combined effect is expected to exceed the individual impacts of the EOs, thereby fostering more sustainable practices in livestock production. Therefore, the objectives of this study were to (1) systematically screen various combinations of five selected essential oils using mixture design methodology, (2) identify optimal blends that can bolster rumen fermentation efficiency, and (3) evaluate the methane mitigation potential of the most promising combinations using in vitro rumen fermentation systems.

## 2. Materials and Methods

### 2.1. Experimental Design and Ethics Statement

This study employed a two-stage in vitro approach to evaluate different EO combinations for rumen fermentation optimization. In Experiment 1, various EO blends were screened using a modified Tilley and Terry [43] batch culture system to identify combinations with optimal fermentation profiles. Based on these results, the most promising combinations were selected for assessment in Experiment 2, which assessed their effects on total gas and CH_4_ production using pressure transducer methodology by Theodorou [44]. Rumen fluid was collected from permanently cannulated, non-pregnant, non-lactating Holstein dairy cows maintained under standard management conditions. This study was conducted with prior authorization from the Animal Experimentation Ethics Committee of the Universitat Autònoma de Barcelona (UAB) and the Competent Authorities of the Generalitat de Catalunya (Ref: DGPAMN 10922). All procedures involving animals complied with the ethical standards outlined in the European Union directives for the care and use of animals in research.

### 2.2. Experiment 1: Optimizing Fermentation Characteristics Through Targeted EO Synergy Analysis

#### 2.2.1. Experimental Treatments and Incubation Substrate

**Figure 1:**
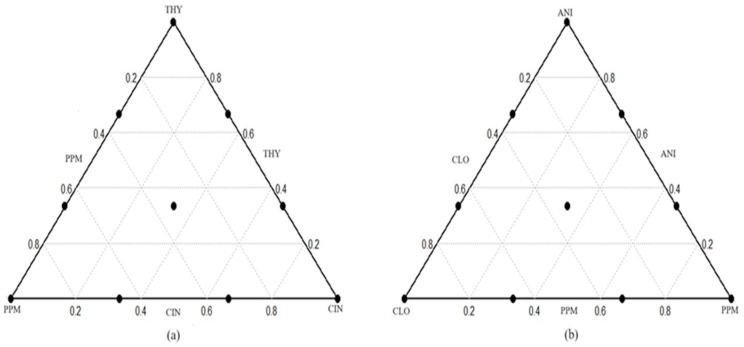
The Simplex Centroid Design is applied to optimize component mixtures. (**a**) THY = thyme oil, PPM = peppermint, CIN = cinnamon leaf oil; (**b**) ANI = anise oil, CLO = clove leaf oil, PPM = peppermint.

Five EOs were provided by Kaesler Nutrition GmbH (Bremerhaven, Germany) and stored at 4 °C in amber glass bottles. The essential oils and their main active compounds are detailed in Table 1. The five essential oils were evaluated using two combination triads based on Simplex Centroid Design (SCD) [45]. Triad 1 tested combinations of thyme oil (THY), peppermint (PPM), and cinnamon leaf oil (CIN), and triad 2 considered possible combinations of anise oil (ANI), clove leaf oil (CLO), and peppermint (PPM). Each triad combination included 10 different mixtures: three individual oils, at the extreme corners, represent pure EOs. Six binary combinations (67:33 and 33:67 ratios) are marked at midpoints of the sides of the triangles, and one ternary mixture (33:33:33) represents the center point of the triangle (Figure 1). In this way, 10 different mixtures were formulated for each of the two-triad combinations and assessed. Each of these mixtures was subjected to triplicate in vitro incubation during each experimental period, with the study being replicated twice (two experimental periods). All treatments were tested at a consistent dose of 400 mg/L. Essential oil solutions were prepared in ethanol (96% *v*/*v*) and added at 0.2 mL per incubation tube [46]. Control and blank treatments received equivalent volumes of ethanol to ensure experimental uniformity.

Table 2 details the composition of the 10 different mixtures assessed for each combination triad (triad 1 and triad 2) used in the study. These mixtures comprised three treatments involving individual EOs, six mixtures composed of two different EOs, and one mixture containing the three EO types for each triad. Each series of experiments also incorporated a negative control (CTR; diet without EO) and a blank consisting of rumen fluid–buffer without diets or EOs. In total, 12 treatments were included in each triad assessment.

The experimental diet, used as the incubation substrate, consisted of 50% alfalfa hay, 20% corn grain, 20% barley grain, and 10% soybean meal. All ingredients were ground to pass through a 1 mm screen sieve (Cyclotec CT 293, Foss, Barcelona, Spain) and thoroughly mixed before use.

#### 2.2.2. In Vitro System

To evaluate the effects of the different treatments on microbial fermentation, including pH, ammonia-N (NH_3_-N), and individual volatile fatty acid (VFA) concentration, we utilized the batch in vitro model procedure of Tilley and Terry [43] with slight modifications aimed at focusing on microbial activity rather than total digestibility. Specifically, only the 24-h rumen fermentation phase was conducted, excluding the pepsin digestion step, which was included in the original method. The inoculum was prepared using a 1:1 (*v*/*v*) ratio of rumen fluid to McDougall’s buffer under N-free O_2_ to maintain anaerobic conditions.

#### 2.2.3. Rumen Fluid Collection

Rumen fluid was collected from cannulated cows at 08:00 h before morning feeding, following an overnight fast (12 h). Approximately 4 L of ruminal contents were collected from the ventral sac of the rumen, strained through four layers of cheesecloth, and transported to the laboratory within 15 min in pre-warmed (39 °C) thermos containers under anaerobic conditions. This ruminal fluid was homogenized before being used for preparing the inoculum media for the two in vitro incubation approaches.

#### 2.2.4. The in Vitro Procedure for Evaluating Rumen Microbial Fermentation

The inoculum medium was prepared by mixing ruminal fluid with McDougall’s buffer [47] in a 1:1 (*v*/*v*) ratio, whose pH was adjusted to 6.6 using HCl (37% *v*/*v*) or 6N NaOH as needed. The inoculum medium was continuously stirred using a magnetic stirrer and maintained at 39 °C under continuous N-free O_2_ flushing to ensure anaerobic conditions.

Incubations were carried out in triplicate, using 100 mL polypropylene tubes containing 50 mL of the incubation medium, 0.5 g of diet, and 0.2 mL of the treatment solution or ethanol. After inoculation, tubes were again flushed with N-free O_2_ gas, sealed with gas-release rubber stoppers, and incubated in a shaking water bath at 39 °C for 24 h.

#### 2.2.5. Sample Collection

After 24 h incubation, pH was measured using a portable digital pH meter (sensION+ Model PH31, HACH Company, Barcelona, Spain). Samples were collected for NH_3_-N and short-chain VFA analysis.

For NH_3_-N determination, a 4 mL 24 h incubated sample was collected and acidified with 4 mL of 0.2 N HCl, then frozen at −20 °C until analysis. After thawing, samples were centrifuged at 15,000× *g* for 15 min at 7 °C, and the supernatant fraction was analyzed by spectrophotometry (Libra S21; Biochrom Technology; Cambridge, UK), as described by Chaney and Marbach [48].

For VFA analysis, samples were systematically pooled by treatment and frozen for analysis. Total and individual VFAs were analyzed using high-performance liquid chromatography (HPLC) with a SHIMADZU system and Agilent 1100 Series, employing an ultrabase C18 chromatographic column (1.8 μm, 150 × 4.6 mm, Zorbax, Agilent, Santa Clara, CA, USA) following the same procedure as indicated by Temmar et al. [49].

### 2.3. Experiment 2 (Optimizing Total Gas and CH_4_ Output Using Targeted EO Combinations)

#### 2.3.1. Experimental Treatments and Incubation Substrate

Based on Experiment 1 results, the two most effective essential oil combinations were selected for further evaluation: T1 (CIN 80% + PPM 20%) and T2 (ANI 80% + CLO 20%). These treatments were compared against a negative control (diet without EO), a positive control (diet with monensin), and a blank (buffer without diet or EO), as mentioned in Table 3. The diet used as the incubation substrate and the rumen fluid collection procedure were the same as those used in Exp. 1. To investigate the effects of selected treatments on total gas and CH_4_ production (including the kinetics), we conducted a pressure transducer-based incubation procedure, following the methodology proposed by Theodorou et al. [44]. Micro-minerals and resazurin were omitted from the procedure, as suggested by Mould et al. [50].

#### 2.3.2. The in Vitro Procedure for Evaluating Total Gas and CH_4_ Production and Kinetics

Before the morning feeding, approximately 1 liter of rumen fluid was obtained from a cannulated non-pregnant, non-lactating dairy cow on the day of each incubation. The collected rumen fluid underwent straining through four layers of cheesecloth and was promptly transported to the laboratory in a pre-warmed thermos container with no headspace. Subsequently, the homogenized ruminal fluid was utilized to prepare the inoculum media essential for the two in vitro incubation methods. The procedure was similar to that described previously for evaluating rumen microbial fermentation, with the following minor variations. (1) The inoculum media were prepared by mixing ruminal fluid with the buffer solution [37] in a 4:1 (*v*/*v*) ratio to prevent issues related to excessive total gas production and accumulation. (2) Incubations were conducted in quadruplicate (two bottles to measure total gas production and the other two bottles for methane determination) in 60 mL serum bottles, with 50 mL of incubation media fluid, 0.5 g of diet, and the corresponding volume (0.2 mL) of the treatment solution or ethanol. (3) Immediately after the inoculation media were dispensed, the tubes were flushed with N-free O_2_ gas, sealed with a butyl rubber stopper and aluminum caps, and incubated for 24 h in a water bath at 39 °C. This incubation period was chosen to capture the peak phase of microbial fermentation and gas production, particularly for rapidly fermentable substrates. Prior studies have demonstrated that the majority of total gas and methane (CH_4_) production occurs within the first 24 h of in vitro incubation, making this duration appropriate for assessing treatment effects on fermentation kinetics [51,52].

#### 2.3.3. Gas Measurements and Analysis

Gas pressure was measured at 1, 2, 4, 6, 8, 12, and 24 h using a pressure transducer (Model HD-8804, DELTA OHM, Padova, Italy). Subsequently, gas volume was calculated using a pre-established linear equation, V = 0.067 + 1.172p, where V represents gas volume (mL) and p represents pressure (kPa).

For CH_4_ analysis, gas samples were collected at the same time points using BD Vacutainer needles and transferred to 12 mL glass vials (Exetainer^®^, Labco, UK). Methane concentration was determined by gas chromatography (GC6890A, Agilent, Santa Clara, CA, USA) equipped with a flame ionization detector and HP-1 capillary column (30.0 m × 530 μm × 1.50 μm). Helium carrier gas flow was maintained at 12 mL/min with an oven temperature of 120 °C.

At the end of the incubation period, all bottles were opened, and pH was determined using a portable digital pH meter (sensION+ Model PH31, HACH Company, Barcelona, Spain).

#### 2.3.4. Statistical Analysis

##### Experiment 1

Data were analyzed using a randomized complete block design with SAS 9.4 (SAS Institute Inc., Cary, NC, USA). The Simplex Centroid Design (SCD) was analyzed using response surface methodology in R software (v. 4.2.1, 2022, R Foundation for Statistical Computing, Vienna, Austria). Treatment effects were compared to controls using Dunnett’s test, with significance declared at *p* < 0.05 and trends at 0.05 < *p* < 0.10.

##### Experiment 2

Gas production kinetics were fitted to the Gompertz model [53] as follows:

Y = a exp {−b exp (−ct)}

where Y is cumulative gas volume (mL) at time t (h); a is the intercept representing gas from the soluble fraction; b is the extent of gas production from the potentially degradable fraction; and c is the fractional rate of gas production.

Model parameters were estimated using the NLIN procedure of SAS with iterative least squares and the Marquardt algorithm. Statistical analysis was performed using PROC MIXED in SAS 9.4, with treatment as a fixed effect and experimental period as a random block effect. Means were compared using Tukey’s multiple comparison test, with significance declared at *p* < 0.05.

## 3. Results

### 3.1. Experiment 1 (Screening the Best Combination for an Optimum Rumen Fermentation)

This experiment was designed to identify specific combinations of EOs that significantly influence the concentration and profile of VFAs and the levels of NH_3_-N. Although the SCD provided a structured framework for modeling essential oil mixtures, the prediction error sum of squares (PRESS) values were relatively high for several outcomes (as shown in Table 4 and Table 5), particularly for binary and ternary combinations. Additionally, some model predictions were unstable or biologically implausible, such as negative values. These limitations are typical in mixture experiments, likely due to limited variability across treatments and overlapping responses among mixtures.

**Figure 2:**
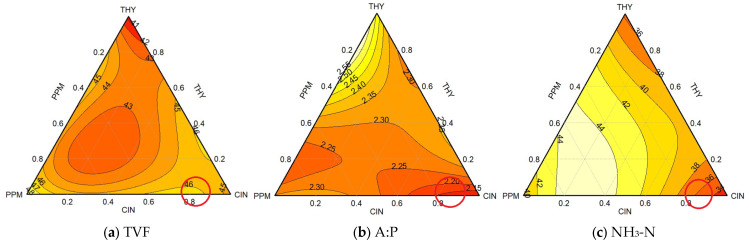
Contour plots (2D) showing the effects of essential oil combinations under triad 1 on (**a**) total volatile fatty acids, (**b**) acetate-to-propionate ratio, and (**c**) ammonia-N levels. (Lines within plots depict zones of constant response). The most optimal trend/values, based on observed results for the combination of EOs within combination triad 1, are marked by red circles.

**Figure 3:**
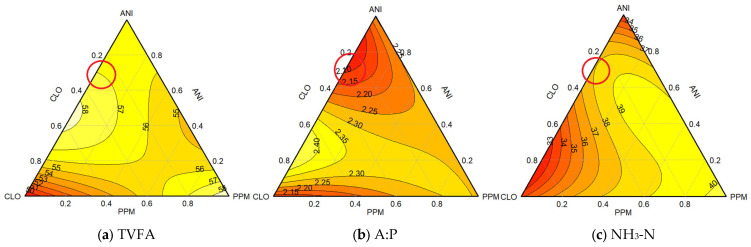
Contour plots (2D) showing the effects of essential oil combinations under triad 2 on (**a**) total volatile fatty acids, (**b**) acetate-to-propionate ratio, and (**c**) ammonia-N levels. (Lines within plots depict zones of constant response). The most optimal trend/values, based on the observed results for the combination of EOs within combination triad 2, are marked by red circles.

Nonetheless, linear terms for pure treatments were well estimated, and overall model fit statistics, such as R^2^ values, remained acceptable in most cases. To enhance interpretability, SAS was used to compile observed treatment means, providing a more reliable comparison of actual responses across all combinations. The analysis revealed that several binary and ternary combinations outperformed the pure treatments in terms of response values. Importantly, contour plots (Figure 2 and Figure 3) served as the primary interpretive tool, visually capturing spatial trends in total VFA concentration, acetate-to-propionate ratio (A:P), and NH_3_-N. These plots effectively illustrated performance differences among EO mixtures, even in cases where the numerical model outputs were less stable.

#### 3.1.1. Combination Triad 1 (Different Proportions of THY, PPM, and CIN EOs)

When used individually, PPM oil clearly produced the highest total VFA (TVFA) concentration, significantly surpassing CIN, which only exhibited a moderate increase. In contrast, THY oils had no significant impact (Table 4 and Table 6). Regarding possible synergistic effects, an increase in TVFAs, attributed predominantly to PPM within an 85–95% range, is depicted in Figure 2a for the 45 mM concentration. The significant coefficient of TVFAs for the subsequent concentration of 46 mM was associated with a blend of CIN and PPM within a 60–85% range, with CIN accounting for up to 85% and PPM contributing 15%. This suggests that synergy occurred in the CIN and PPM blend that increased TVFA concentration.

Regarding the impact of single EO on the molar proportions of VFAs, propionate was only increased by CIN alone, while THY resulted in the most significant rise in butyrate molar proportions. Moreover, the lowest A:P was observed for CIN, whereas THY had no effect. The two-dimensional contour plot for A:P (Figure 2b) revealed the minimal ratio value of 2.10 within an 80–100% range, predominantly influenced by CIN. The next best ratio of 2.20 was linked to the synergistic effect of CIN and THY, spanning a 65–100% range, with CIN contributing up to 95% and THY contributing the remaining 5%. The highest A:P ratio of 2.55 was attributed to the combined effect of THY and PPM within a 05–35% range, with THY as the primary influencer.

Concerning the effect of each EO on NH_3_-N concentration (mg/100 mL), the lowest concentration was recorded for CIN (Table 6). Figure 2c depicts CIN as significantly associated with the minimal NH_3_-N concentration of 34 mg/100 mL. The second-lowest concentration of 36 mg/100 mL was ascribed to a blend of 85% CIN and 15% PPM.

To identify a combination leading to enhanced results, characterized by a higher TVFA concentration, a lower A:P ratio, and a reduced NH_3_-N concentration, an integrated analysis of all triangle plots was undertaken. The overarching trends are summarized in Table 7. The red circles within the triangle plots (Figure 2) represented the most significant findings. The selection of the best combination required an examination of the overall trend rather than a singular focus on the highest value for a specific response variable. For instance, among the given trends (Table 7), the highest TVFA value of 48 mM is attributed predominantly to the effect of PPM. However, this trend is not promising because, despite having the highest TVFA value, it contrasts with an elevated NH_3_-N concentration of 40 mg/100 mL and a higher A:P ratio of 2.30 among all the trends. The optimal combination within the existing EO blend was delineated by results showing 46 mM for TVFAs, 2.20 for the A:P ratio, and 37 mg/100 mL for NH3-N, achieved through a mixture of 80% CIN and 20% PPM.

#### 3.1.2. Combination Triad 2 (Different Proportions of ANI, CLO, and PPM EOs)

Within the framework of the SCD treatments, the highest concentration of TVFAs was observed in PPM, which exhibited statistically significant differences when compared to the CTR, as detailed in Table 8. The peak TVFA value of 58 mM, predominantly attributed to the influence of PPM within a 95–100% range, is illustrated in Figure 3a. Another significant observation was a TVFA value of 58 mM within a 40–65% range, mainly from the use of CLO with minimal involvement of ANI.

Among individual EOs used, the lowest A:P ratio was recorded for CLO, followed by ANI and PPM (Table 5 and Table 8), with only CLO reaching a statistically significant difference when compared to the CTR, as indicated in Table 8. The minimal A:P ratio of 2.10, significantly impacted by ANI (90%) with a minor contribution from CLO (10%), is highlighted in the corresponding contour plot (Figure 3b). A synergistic effect leading to an A:P ratio of 2.15 was observed in a mixture comprising 80% ANI and 20% CLO.

In terms of NH_3_-N concentrations, the lowest value was noted for ANI, followed by CLO and PPM (Table 5 and Table 8), reaching only ANI and CLO and showing significant differences when compared to the CTR, as presented in Table 8. Figure 3c shows a combination of ANI and CLO resulting in the lowest NH_3_-N concentration of 33 mg/100 mL within a 70–90% range, with CLO making a significant contribution (70%) alongside ANI (30%). The second-lowest value of 34 mg/100 mL was achieved with a mixture of 95% ANI and 5% CLO.

A comprehensive analysis of all triangle plots was conducted to discern the most advantageous trend and ascertain the optimal EO combination, as shown in Table 9. The selection of any combination as optimal is based on its alignment with key performance indicators observed in the results. Similar to what was observed for combination triad 1, the most favorable value is marked by red circles in Figure 3. The peak TVFA value of 58 mM, assessed in conjunction with the corresponding NH_3_-N concentration, shows a level of 40 mg/100 mL. Further analysis reveals an A:P ratio of 2.30, the highest observed among trends. This trend is driven by the influence of PPM (90%) and CLO (10%). The higher A:P ratio indicates a shift toward less favorable fermentation conditions. The trend was significantly impacted by ANI (80%), with a minor contribution from CLO (20%), characterized by a TVFA of 57 mM, an A:P ratio of 2.10, and an NH_3_-N level of 38 mg/100 mL, indicating a favorable balance for efficient fermentation.

### 3.2. Experiment 2 (Total Gas and Methane Production)

In Experiment 1, two EO mixtures were selected for further evaluation of their effects on total gas and CH_4_ production, following their significant impact on key rumen fermentation parameters. The first mixture, comprising PPM and CIN (80:20) (T1), and the second mixture, a blend of ANI and CLO (80:20) (T2), exhibited notable effects in previous trials. Due to their promising potential to modulate rumen fermentation, these two combinations were selected for detailed analysis in Experiment 2, specifically focusing on their impact on total gas production and methane emissions.

**Figure 4:**
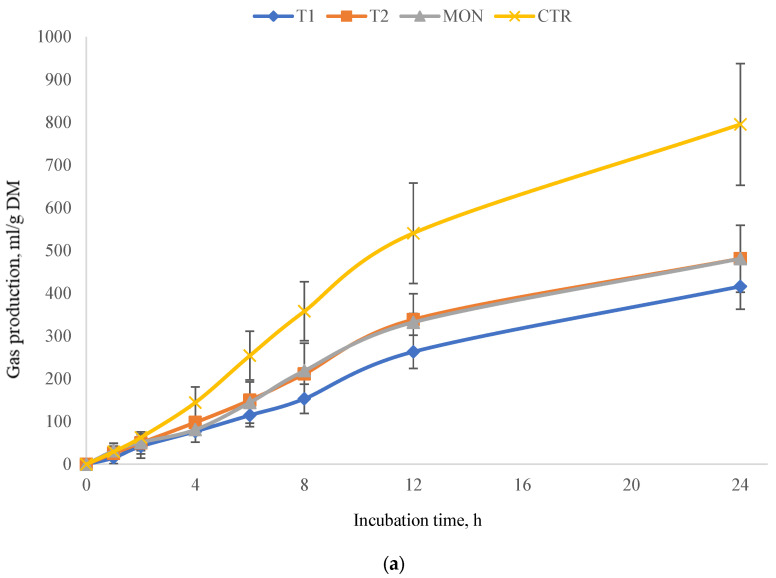

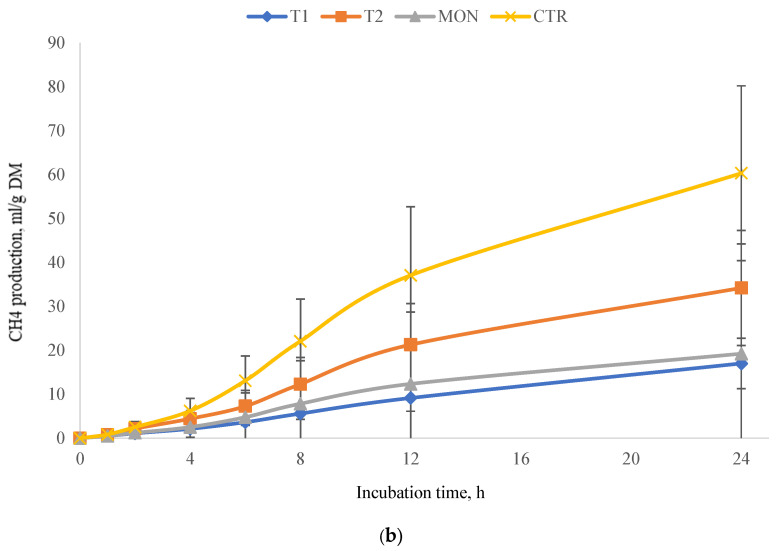
Effects of different treatments on total gas and methane production at different incubation times (error bars indicate standard deviation). (**a**) shows the cumulative gas production pattern of different treatments incubated. (**b**) shows the cumulative CH_4_ production pattern of different treatments incubated. Treatments: CTR = 50:50 F:C diet as substrate; MON = monensin as a positive CTR; T1 = CIN 80% + PPM 20%; T2= ANI 80% + CLO 20%.

The results of the total gas kinetic constants for all the treatments and graph representation of the gas production pattern are presented in Table 10 and Figure 4a, respectively. No significant difference (*p* > 0.05) was observed in the regression coefficients a and b of the gas production kinetics between different experimental treatments. However, the rate constant of gas production (c) was highly significant (*p* < 0.01), and it was lower for the first mixture compared to the other treatments.

Fitting the methane production data with the Gompertz model [53] was attempted; however, the model did not adequately represent the data due to the absence of a clear sigmoidal trend in the production curves. Consequently, fermentation kinetics associated with methane were excluded from the analysis. However, graphical representations of the methane production pattern are presented in Figure 4b. The high variability in methane production among treatments is visually evident from the standard deviation bars included in the graph.

The results of cumulative total gas and CH_4_ production in 24 h, the ratio CH_4_/total gas, and pH after 24 h of incubation are indicated in Table 11. According to the results, all treatments, including the positive control (MON), significantly influenced total gas and CH_4_ production compared to the control group (CTR) (*p* < 0.05). The MON, used as a positive control, significantly affected the total gas and CH_4_ production and the ratio CH_4_/total gas compared to the CTR. Notably, T1 resulted in the lowest cumulative total gas and CH_4_ production (*p* < 0.05) and the lowest ratio CH_4_/cumulative gas (*p* < 0.05), similar to the MON. The effect of T2 was also significant (*p* < 0.05) on total gas and CH_4_ production, yet it displayed the highest CH_4_/total gas ratio, which did not differ significantly from that of the CTR. In terms of pH levels, all treatments significantly influenced pH (*p* < 0.01); however, the observed differences were of minimal biological significance.

To further explore the effect of EO combinations on CH_4_, values were also predicted based on VFA profiles using the empirical formula by Foiklang et al. [54]. Table 12 compares these predicted values with actual observed CH_4_ outputs. For T1, the observed CH_4_ was lower than predicted, while T2 showed the opposite trend. Monensin and control treatments were excluded from this comparison, as their CH_4_ mitigation mechanisms differ mechanistically and may not be accurately captured by VFA-based prediction models.

## 4. Discussion

### 4.1. Experiment 1 (Rumen Microbial Fermentation)

EOs were categorized into two distinct groups based on their potential interactions with different types of active compounds [55]. Each category comprises two phenolic EOs (THY & CIN in triad 1; ANI & CLO in triad 2) and one terpene EO (PPM, a monoterpenoid alcohol). This classification aims to harness their complementary and synergistic effects in the regulation of rumen fermentation conditions. Other authors have described how, when used together, they significantly enhance feed efficiency and overall animal performance in cattle diets [56,57]. The results of this study demonstrated that while individual EOs, such as CIN and PPM, exhibited superior performance in specific parameters (e.g., TVFA concentration and A:P ratio, respectively), the synergistic blends consistently provided more balanced and optimal outcomes across multiple response variables, including TVFA concentration, A:P ratio, and NH_3_-N concentration.

#### 4.1.1. Individual Efficacy: Baseline Performance of Essential Oils

In our study, individual EOs significantly affected specific ruminal fermentation parameters. For instance, in both combination strategies, PPM resulted in the highest TVFA production. This can be attributed to its unique bioactive compound, menthol, which selectively inhibits gram-positive bacteria and protozoa while promoting the proliferation of VFA-producing microbes [58]. Patra and Yu [59] reported that peppermint oil, administered at a concentration of 500 mg/L in an in vitro system, significantly elevated TVFA production by selectively suppressing methanogens and enhancing the activity of VFA-producing bacteria. Our findings resonate with previous research. For example, Farghaly and Abdullah [60] reported that peppermint oil supplementation increases ruminal VFAs, indicating its potential to modulate rumen fermentation favourably. Conversely, Hosoda et al. [61] reported that supplementation of PPM in dairy cows did not elicit any significant changes in TVFA production, suggesting that in vivo responses may differ from in vitro observations.

When used individually, CIN improved the A:P ratio and reduced NH_3_-N levels but did not maximize TVFA production to the same extent as PPM. These results align with the work of Castillejos et al. [26], who reported that CIN improved propionate production and nitrogen efficiency but cautioned that its benefits might not be fully realized without complementary mechanisms of action. These findings align with our results, highlighting the limitations of single essential oil applications and supporting the need for a more synergistic approach. Individually applied essential oils can exert strong antimicrobial effects, potentially inhibiting microbial fermentation and substrate degradation. This has been observed in long-term fermentations by Spadini et al. [62] and further supported by Simoni et al. [63], who reported similar disruptions in microbial activity.

Similarly, CLO significantly influenced propionate levels and the A:P ratio; this effect is consistent with findings by Saeed et al. [64] and Benchaar et al. [65], who reported that eugenol, a major component of clove oil, can shift fermentation toward propionate production by selectively inhibiting acetate-producing microbes. Supporting this assertion, Castillejos et al. [66] also observed that clove oil supplementation increased propionate molar proportions in vitro.

Our research further revealed that ANI profoundly impacted NH_3_-N concentrations. The significant reduction in NH_3_-N concentrations with ANI supplementation could be due to ANI’s potential to inhibit the activity of hyper-ammonia-producing bacteria (HAPB) or to alter protein degradation pathways in the rumen. This is consistent with the findings of Chahaardoli et al. [67], who reported that anise oil decreased ammonia production in an in vitro study. The mechanism appears to involve the disruption of cell membranes in certain bacterial populations, leading to their death and reduced urease enzyme activity, which converts urea into ammonia [68]. It also lowers populations of ammonia-producing and proteolytic bacteria [69] and inhibits amino acid breakdown [42]. Additionally, it decreases protozoa populations, which are essential for breaking down proteins into ammonia.

In this study, THY showed limited effectiveness, which suggests that its antimicrobial and fermentation-modulating properties may not be as robust or consistent as those of other EOs, particularly within the tested combinations. These results contrast with findings from Ribeiro et al. [70], who noted that supplementing high-forage diets with 1.25 g/kg dry matter (DM) of thyme EO enhanced ruminal fermentation by increasing the molar proportions of propionate and butyrate while reducing the A:P ratio. In contrast, our findings align with those of Benchaar [71], who determined that thyme EOs did not significantly affect fermentation parameters, potentially due to microbial adaptation or an insufficient concentration of active compounds. The variability in thyme’s effects across different studies indicates that its efficacy may depend on factors such as dosage, dietary composition, and microbial community structure [26,30]. This underscores the necessity for tailored EO formulations rather than one-size-fits-all applications in ruminant nutrition.

To ensure optimal rumen function, it is vital to effectively balance multiple fermentation parameters [72]. Merely concentrating on maximizing a single aspect can lead to suboptimal fermentation efficiency. For instance, our research has shown that relying on ANI alone is counterproductive; lower NH_3_-N was found alongside a lower TVFA level and an unfavorable A:P ratio. This emphasizes the significant trade-offs linked to single EO applications. Additionally, our findings echo the study conducted by Lin et al. [73], which revealed that improper use of certain EOs could disrupt the delicate balance of rumen fermentation. Therefore, it is crucial to recognize the limitations of single EO applications and adopt a more integrated approach for optimal results.

#### 4.1.2. Enhanced Efficacy: Synergistic Outcomes of Essential Oil Combinations

By focusing on trends and interactions rather than individual factors, the CIN + PPM blend emerged as the optimal combination, exhibiting a strong synergy that enhanced TVFA production while maintaining a favorable A:P ratio and reducing NH_3_-N concentration. PPM likely enhanced microbial activity and VFA production, and CIN improved nitrogen utilization by inhibiting HAPB, thereby reducing NH_3_-N concentrations. As proposed by McIntosh et al. [74], CIN disrupts the cell membrane integrity of Gram-negative bacteria, reducing proteolytic activity and leading to lower NH_3_-N accumulation.

The observed synergy between CIN and PPM is further explained by their distinct chemical nature. CIN, a phenolic compound, is known for its potent antimicrobial properties, selectively inhibiting methanogenic archaea and proteolytic bacteria, thereby enhancing VFA production and reducing methane emissions [30,75]. PPM, rich in terpenes, complements CIN by improving its bioavailability and penetration into microbial cells while providing mild antimicrobial and antioxidant effects that support overall microbial balance [65,76]. This combination of phenol and terpene-rich compounds may create a balanced and effective fermentation profile, as demonstrated by the improved TVFA production, reduced A:P ratio, and lower NH_3_-N levels observed in our study.

Our results align with those reported by Sallam et al. [77], where a blend of EOs, including CIN and PPM, led to a 15–20% reduction in NH_3_-N concentration compared to the control group. Furthermore, the VFA profile in their study exhibited a 10–12% increase in propionate proportion, along with a decrease in the A:P ratio, reflecting a shift towards more efficient energy utilization. The reduction in NH_3_-N concentration observed in our study is particularly significant, as it suggests improved nitrogen retention and reduced nitrogen waste, which is critical for sustainable livestock production. This synergistic reduction in NH3-N aligns with previous studies that have reported the potential of EO blends to mitigate nitrogen losses and improve nutrient utilization [78,79].

In contrast to the work of Cardozo et al. [46], who found that some EOs, when used alone, could disrupt microbial balance or lead to inconsistent fermentation outcomes, our findings demonstrate that blending EOs can overcome these limitations. For example, while PPM alone resulted in the highest TVFA concentration but a suboptimal A:P ratio and elevated NH_3_-N levels, the combination of CIN and PPM achieved a balanced improvement across all parameters. This suggests that the negative effects observed by Cardozo et al. [46] may be mitigated through the synergistic effects of EO blends, as demonstrated in our study.

A comprehensive evaluation revealed that the combination of PPM at 90% and CLO at 10% yielded the highest TVFA production but also increased A:P ratios and NH_3_-N levels. This suggests that, while beneficial, the combination does not achieve an optimal fermentation profile. The higher A:P ratio is likely due to PPM’s strong inhibitory antimicrobial effects on propionate-producing bacteria, such as *Megasphaera elsdenii* and *Selenomonas ruminantium*. This inhibition reduces propionate levels, leading to an increase in the A:P ratio, which is generally considered less favourable, as it indicates a shift toward acetate-dominated fermentation, which is less energetically efficient compared to propionate production. This aligns with the findings of Patra and Yu [59], who noted that certain EOs could disrupt microbial populations, leading to imbalances in fermentation end products.

Similarly, the increased NH_3_-N levels suggest incomplete utilization of nitrogen, likely due to microbial inhibition or altered proteolytic activity, which Wallace et al. [80] linked to excessive EO antimicrobial activity impairing nitrogen metabolism. This indicates inefficient incorporation of nitrogen into microbial biomass, resulting in potential losses and reduced fermentation efficiency. These findings suggest that the combination of these two EOs did not result in a synergistic improvement across all parameters. Instead, the less desirable traits of PPM, such as its broad-spectrum antimicrobial activity, appeared to dominate in the blend, overshadowing the beneficial effects of CLO. This trend indicates a potential imbalance in nutrient assimilation, pushing the fermentation profile beyond optimal conditions and hinting at reduced efficiency in overall system stability.

Studies on phenolic combinations in EOs have shown that their synergistic effects can increase the efficacy of antimicrobial and antioxidant activities, particularly in inhibiting pathogenic microorganisms and reducing oxidative stress in biological systems [56,81]. The combination of ANI (80%) and CLO (20%), both rich in phenolic compounds, demonstrates a harmonious and synergistic interaction in modulating ruminal fermentation. The resulting lower A:P ratio of 2.10 suggests well-regulated nutrient conversion, while the stable levels of TVFA and N-NH_3_ indicate efficient metabolic activity without excessive accumulation of by-products. Together, these metrics suggest an optimal alignment that supports sustained nutrient assimilation and system stability, making this combination particularly advantageous. The findings align with those of Vasta et al. [82], who demonstrated that phenolic compounds could enhance propionate production while reducing ammonia levels, underscoring their potential for optimizing rumen function.

The phenolic compounds, such as anethole in ANI and eugenol in CLO, respectively, contribute significantly to these observed effects. Known for their antimicrobial properties, these compounds influence rumen microbial populations and metabolic pathways through several mechanisms. These include disrupting microbial cell membranes, inhibiting essential enzyme activity, and modulating the composition and activity of microbial communities. These actions are crucial for maintaining efficient and stable fermentation processes within the rumen.

Anethole has been shown to inhibit proteolytic bacteria, such as Clostridium and Bacteroides, reducing proteolysis and deamination and thereby lowering NH_3_-N levels. This improves nitrogen utilization efficiency, as supported by Cardozo et al. [83] and Fandiño et al. [84], who found that phenolic compounds can reduce ammonia production by inhibiting proteolytic activity. On the other hand, CLO, rich in eugenol, selectively inhibits methanogens and acetate-producing bacteria while promoting propionate-producing species, such as *Selenomonas ruminantium*. This shift toward propionate is energetically favourable, as propionate serves as a key precursor for gluconeogenesis in ruminants. The work of Bokharaeian et al. [85] supports this, showing that eugenol-rich EOs can improve the A:P ratio by redirecting metabolic pathways toward propionate synthesis.

ANI and CLO collaborate effectively by utilizing their shared phenolic properties to create a balanced fermentation profile. ANI reduces NH_3_-N levels, while CLO promotes propionate production, leading to a more efficient and stable fermentation environment. This combination showcases the potential of phenolic-rich EOs to optimize ruminal fermentation without significant trade-offs. Importantly, this synergy helps avoid the imbalances often observed in other EO blends, where one EO may dominate and produce unfavourable outcomes.

### 4.2. Experiment 2 (Total Gas and Methane Production)

This study’s initial screening decisively identified two EO combinations for further exploration: T1 (CIN 80% + PPM 20%) and T2 (ANI 80% + CLO 20%). These combinations were chosen for their significant synergistic effects, demonstrating the clear transformative potential of synergy in EO formulations. From a practical standpoint, these findings suggest that pre-formulated EO blends should be designed based on mechanistic interactions rather than empirical selection alone. This could enhance the efficiency of natural feed additives in improving rumen function, reducing nitrogen waste, and mitigating GHG emissions.

Total gas and CH_4_ production are key indicators of the presence of degradable carbohydrates in the rumen, particularly cellulose, which can be digested. Research by Molho-Ortiz et al. [86] indicates that levels of these gases are positively correlated with the production of VFAs. In our study, we found that treatments T1 and T2 were successful in reducing CH_4_ and gas production without negatively influencing the overall or individual concentrations of VFAs. This finding suggests that these treatments selectively target methanogenic archaea while preserving the overall rumen microbial community and fermentation processes.

Our results align with the work of Brice et al. [87], who found that EO blends (EOB) reduced total gas production when compared to a control group, noting variations in cumulative gas production among the different EOB treatments. Similarly, Lin et al. [88] investigated the effects of a blend of thyme, oregano, and cinnamon essential oils on in vitro ruminal fermentation, reporting a significant reduction in total gas production and CH_4_ emissions. Their study also noted a decreased A:P ratio and NH3-N production, indicating enhanced fermentation efficiency. Additionally, Temmar [89] evaluated the impact of a combination of Anise and Cassia essential oils on rumen microbial activity and gas production, observing a reduction in total gas production and CH_4_ emissions. Their findings also highlighted that this reduction occurs without decreasing TVFAs. Together, these studies provide robust evidence that EOBs can effectively modulate ruminal fermentation, reduce methane production, and enhance nutrient utilization.

T1 exhibited substantial CH_4_ mitigation that was largely driven by the bioactive properties of CIN (cinnamon leaf oil, predominantly rich in eugenol at ~80%, a phenolic compound, with minimal cinnamaldehyde content as an aldehyde), with some contribution from PPM. Our results align with El-Azrak et al. [90], who reported that EO blends incorporating thyme, cinnamon, and peppermint led to a reduction in methane emissions of up to 25% in an in vitro rumen fermentation model.

The CIN used in this study has well-documented inhibitory effects on methanogenesis, primarily through eugenol’s interference with archaeal enzyme activity [75,91] and its ability to alter rumen microbial composition [59]. While Macheboeuf et al. [92] reported that pure cinnamaldehyde (the main component of cinnamon bark oil, not leaf oil) reduced methane production by 94% at 5 mM concentration, the eugenol-rich CIN used in our study demonstrates comparable anti-methanogenic properties through different mechanisms. This effect is complemented by menthol from PPM, which enhances cell membrane permeability and indirectly influences microbial metabolism, thereby amplifying the bioavailability and effectiveness of eugenol and other bioactive compounds in CIN. Similar synergistic outcomes were observed by Benchaar et al. [93], who noted that menthol’s ability to enhance membrane permeability could potentiate the activity of other active compounds.

The findings of this study reveal the novel potential of this combination, showing that menthol not only supports cinnamaldehyde’s activity but may also amplify its bioavailability and effectiveness, resulting in a synergistic effect that exceeds the efficacy of either compound alone. These results are in agreement with prior studies on phenolic and aldehyde compounds, which reported CH_4_ reductions of 60% to 70% under specific conditions [31,94] but extend the current understanding by highlighting the critical role of menthol in enhancing bioavailability and efficacy.

T2 demonstrates a novel and complementary mechanism of action, with anethole and eugenol working synergistically to disrupt methanogenic processes. Eugenol’s ability to damage archaeal cell membranes, as demonstrated by McIntosh et al. [74] and Cardozo et al. [83], combined with anethole’s inhibitory effects on hydrogenotrophic methanogens, creates a dual-action mechanism that significantly enhances CH_4_ reduction. These findings are comparable to Castillejos et al. [26], who reported similar reductions using other phenolic compounds in combination, though their effects on fermentation profiles varied. Furthermore, the results align with Kamra et al. [95], who demonstrated the effectiveness of combining phenolic and aromatic compounds in inhibiting methanogenesis, and Wanapat et al. [96], who emphasized the role of EOB in improving rumen fermentation while reducing CH_4_ emissions. While the individual effects of clove and anise oils have been previously documented [97,98], this study is among the first to highlight their synergistic potential when used in combination.

The combination of eugenol and anethole in T2 effectively targeted methanogens, yet their contributions may not be as synergistic as the combination of compounds in T1, which includes cinnamaldehyde and menthol. Although eugenol has a focused action against methanogens by disrupting their cell walls and inhibiting key enzymes necessary for methane production, anethole’s broader spectrum of antimicrobial activity might lead to a less targeted inhibition of methanogenesis [99]. As suggested by Castro-Montoya et al. [100], this broader action could potentially affect other beneficial microbial populations responsible for fiber digestion, thereby diminishing its overall efficacy in methane mitigation. Therefore, careful dose optimization is needed to avoid antagonistic effects on beneficial microbial populations. This could explain why T2, while still effective, did not achieve the same level of methane reduction as T1.

Moreover, the comparison between predicted and observed CH_4_ values revealed notable discrepancies, where T1 produced substantially less CH_4_ than predicted and T2 yielded more. Since the Foiklang et al. [54] model estimates CH_4_ based solely on VFA proportions, these inconsistencies suggest that the essential oil combinations may modulate methane production through additional pathways beyond fermentation shifts. This may involve direct inhibition of methanogens, altered hydrogen availability, or shifts in microbial communities, as similarly reported by Patra & Yu [101], who found that EOs can disrupt methanogenesis by targeting hydrogen-producing microbes and redirecting metabolic hydrogen toward alternative pathways, thereby lowering CH_4_ emissions, even in the absence of major changes in VFA profiles [31]. Furthermore, EOs have been shown to alter rumen microbial composition, including significant shifts in bacterial taxa, such as *Prevotella* and *Clostridia* [102]. These complex, multi-pathway effects highlight the limitations of relying solely on VFA-based models to predict methane output and support the need for a more holistic evaluation of EO efficacy in mitigating ruminal methane.

In this study, the kinetic constant ‘c’, representing the later stages of fermentation, showed statistical significance for both EO combinations (T1 and T2), while ‘a’ and ‘b’ did not. This indicates that the EO blends primarily influenced the later phases of gas production. This delayed effect suggests that the EOs impact microbial activity and substrate utilization as fermentation progresses, particularly during the slower, later phases of gas production. This aligns with findings by Newbold et al. [27], who observed that while overall gas production might not be significantly altered, EOs can modify the kinetics of gas production, especially in later stages. Similarly, Castillejos et al. [26] reported selective impacts of EOs on microbial populations, with those involved in later fermentation stages exhibiting greater sensitivity. The observed delayed influence is also consistent with Patra and Yu [59], who highlighted that EOs often exert a more pronounced influence on microbial activity during the extended phases of fermentation, thereby affecting the later gas production kinetics.

This study advances our understanding of EO synergy in ruminant methanogenesis mitigation by demonstrating that effective blends are not merely additive but result from dynamic interactions that strategically target diverse microbial populations and fermentation pathways. By emphasizing a balanced approach across multiple variables, rather than focusing solely on maximizing single parameters, like TVFAs, or minimizing methane emissions, the research reveals that optimal fermentation efficiency hinges on a holistic system function. The findings highlight the critical role of tailored EO formulations, showcasing that specific combinations, such as CIN+PPM and ANI+CLO, exhibit strong synergistic effects, unlike THY, which showed minimal impact. This novel perspective challenges conventional assumptions, underscoring that targeted EO blending, rather than arbitrary mixing, is essential for achieving sustainable CH_4_ mitigation and enhanced nutrient utilization in ruminant systems.

While the results are promising, several limitations warrant consideration. This study was conducted using an in vitro batch culture system, which, although valuable for controlled screening of fermentation dynamics, does not fully mimic the complexity of the rumen environment in vivo [103]. The absence of structural features such as the rumen wall, the lack of natural stratification of feed particles, and the exclusion of host–microbe interactions limit the extent to which in vitro systems can accurately represent the native ruminal microbial community [104]. Furthermore, essential physiological processes—such as continuous saliva buffering, digesta turnover, microbial absorption, and host–animal feedback—are absent in vitro. These differences may influence microbial activity and fermentation outcomes, including total gas, methane, and VFA profiles, and may limit the direct translation of the findings to live animal systems [105]. Additionally, real-world dietary components, such as fiber content and protein levels, can interact with essential oils (EOs) and potentially modify their efficacy in vivo. Another concern is the potential for microbial adaptation over time; prolonged exposure to these compounds may result in shifts within microbial populations, which could ultimately reduce their effectiveness [63]. Moreover, it is essential to explore dose-dependent effects, as different concentrations of EOs within a blend may influence their interactions. Future research should aim to validate these findings in vivo and investigate strategies to enhance the stability and cost-effectiveness of EOB.

## 5. Conclusions

The findings presented herein provide substantial evidence for the strategic application of EO combinations as a sustainable method for bolstering ruminant productivity without compromising fermentation dynamics. Notably, the combination of CIN + PPM demonstrated the strongest synergy, significantly reducing methane emissions while maintaining volatile fatty acid production. This dual benefit highlights its potential for both environmental sustainability, through reduced greenhouse gas emissions and optimal nutritional performance. Furthermore, the combination of ANI + CLO showed promising synergistic effects, warranting further investigation.

The study demonstrates that effective EO blends are not merely additive but result from dynamic interactions that strategically target diverse microbial populations and fermentation pathways. The research emphasizes that optimal fermentation efficiency requires a balanced approach across multiple variables rather than focusing solely on individual parameters. These findings challenge conventional approaches and underscore that targeted EO blending, based on mechanistic understanding rather than empirical selection, is essential for achieving sustainable methane mitigation and enhanced nutrient utilization in ruminant systems.

However, future research should focus on refining dosages, assessing long-term microbial responses, and evaluating practical applications in livestock feeding to fully utilize the potential of EO synergy in ruminant nutrition. Additionally, in vivo validation studies are needed to confirm these in vitro findings under practical feeding conditions.

## Figures and Tables

**Table 1:** Description of the different essential oils and their main active compounds used in various combinations in Experiment 1.

Product	Main Active Compound (Purity, %) ^1^
Anise oil (ANI)	Trans-anethole (95)
Thyme oil (THY)	Thymol and carvacrol (35)
Cinnamon leaf oil (CIN)	Eugenol (80); cinnamic aldehyde (2.5)
Peppermint oil (PPM)	Menthol (50)
Clove leaf oil (CLO)	Eugenol (84.9)

^1^ Information provided by Kaesler Nutrition GmbH.

**Table 2:** Composition of the main active compounds of essential oils tested in triads 1 and 2.

Treatment ^1^	Proportion of the Main Active Compound in the Mixture, %					
	Triad 1 (%)			Triad 2 (%)		
	THY	PPM	CIN	ANI	CLO	PPM
T1 (100-0-0%)	100	0	0	100	0	0
T2 (67-33-0%)	67	33	0	67	33	0
T3 (33-67-0%)	33	67	0	33	67	0
T4 (0-100-0%)	0	100	0	0	100	0
T5 (67-0-33%)	67	0	33	67	0	33
T6 (33-33-33%)	33	33	33	33	33	33
T7 (0-67-33%)	0	67	33	0	67	33
T8 (33-0-67%)	33	0	67	33	0	67
T9 (0-33-67%)	0	33	67	0	33	67
T10 (0-0-100%)	0	0	100	0	0	100

^1^ Essential oils in triad 1 (THY = thyme oil, PPM = peppermint oil, CIN = cinnamon leaf oil) and triad 2 (ANI = anise oil; CLO = clove leaf oil; PPM = peppermint oil).

**Table 3:** Relative concentrations of the primary active constituents in the essential oils, evaluated in Experiment 2.

EO Treatment	Proportion of the Main Active Compound of Each EO Treatment					Dose, mg/L
	PPM	CIN	ANI	CLO	MON	
T 1	20	80	–	–	–	400
T 2	–	–	80	20	–	400
Monensin	–	–	–	–	100	12.5

EO: PPM = peppermint oil; CIN = cinnamon leaf oil; ANI = anise oil; CLO = clove leaf oil; MON = monensin.

**Table 4:** Interaction model results from the Simplex Centroid Design evaluating the effects of thyme (THY), peppermint (PPM), and cinnamon leaf (CIN) oils on TVFAs, VFA molar proportions, and ammonia-N concentration. Predicted values are presented for individual EOs, binary (50:50), and ternary (33:33:33) combinations. Regression coefficients (Coef), standard errors (SEM), *p*-values, and prediction error sum of squares (PRESS) are included.

Combination of EO in the Mixture	TVFAs ^1^ (mM)			Acetate %			Propionate %			Butyrate %				A:P Ratio ^2^		NH_3_-N ^3^		
	Coef	SEM	*p*-Value	Coef	SEM	*p*-Value	Coef	SEM	*p*-Value	Coef	SEM	*p*-Value	Coef	SEM	*p*-Value	Coef	SEM	*p*-Value
THY	40.5	3.70	<0.01	45.5	1.01	<0.01	18.4	0.92	<0.01	29.6	0.99	<0.01	2.38	0.09	<0.01	35.6	1.94	<0.01
PPM	48.4	3.70	<0.01	44.7	1.01	<0.01	19.4	0.92	<0.01	27.4	0.99	<0.01	2.30	0.09	<0.01	35.7	1.94	<0.01
CIN	44.5	3.70	<0.01	45.0	1.01	<0.01	21.4	0.92	<0.01	26.1	0.99	<0.01	2.10	0.09	<0.01	32.7	1.94	<0.01
THY + PPM	2.86	16.7	0.87	3.46	4.60	0.46	1.04	4.20	0.80	−4.56	4.47	0.33	0.05	0.44	0.91	25.7	8.80	<0.01
THY + CIN	11.4	16.7	0.51	−0.99	4.60	0.83	−2.03	4.20	0.63	0.84	4.47	0.85	0.11	0.44	0.80	19.9	8.80	0.02
PPM + CIN	−2.86	16.7	0.87	7.71	4.60	0.12	1.54	4.20	0.72	−3.93	4.47	0.40	0.20	0.44	0.65	28.4	8.80	<0.01
THY + PPM + CIN	−83.6	124	0.51	55.3	34.0	0.13	42.2	31.1	0.20	−4.90	3.90	0.20	−1.55	3.30	0.64	−3.40	5.10	0.60
Statistical Values																		
RSD	5.23			1.44			1.32			1.39				0.14			6.74	
R^2^	0.99			0.99			0.99			0.99				0.99			0.97	
Adjusted R^2^	0.99			0.99			0.99			0.99				0.99			0.97	
Predicted R^2^	0.97			0.99			0.99			0.99				0.99			0.96	
PRESS	1093			82.9			69.1			78.1				0.78			5955	

^1^ Total volatile fatty acids; ^2^ acetate-to-propionate ratio; ^3^ NH_3_-N (mg/100 mL).

**Table 5:** Interaction model results from the Simplex Centroid Design evaluating the effects of anise oil (ANI), clove leaf oil (CLO), and peppermint (PPM) oils on TVFAs, VFA molar proportions, and ammonia-N concentration. Predicted values are presented for individual EOs, binary (50:50), and ternary (33:33:33) combinations. Regression coefficients (Co-ef), standard errors (SEM), *p*-values, and prediction error sum of squares (PRESS) are included.

Combination of EO in the Mixture	TVFAs ^1^ (mM)			Acetate %			Propionate %			Butyrate %			A:P Ratio ^2^			NH_3_-N ^3^		
	Coef	SEM	*p*-Value	Coef	SEM	*p*-Value	Coef	SEM	*p*-Value	Coef	SEM	*p*-Value	Coef	SEM	*p*-Value	Coef	SEM	*p*-Value
ANI	56.2	2.42	<0.01	46.9	1.26	<0.01	20.9	1.46	<0.01	20.9	1.02	<0.01	2.26	0.19	<0.01	32.7	2.51	<0.01
CLO	48.9	2.42	<0.01	46.2	1.26	<0.01	21.9	1.46	<0.01	22.7	1.02	<0.01	2.13	0.19	<0.01	33.7	2.51	<0.01
PPM	58.8	2.42	<0.01	45.4	1.26	<0.01	19.9	1.46	<0.01	22.9	1.02	<0.01	2.30	0.19	<0.01	40.6	2.51	<0.01
ANI–CLO	22.5	10.9	0.07	4.38	5.70	0.46	−0.47	6.61	0.95	−1.10	4.61	0.81	0.33	0.85	0.71	10.6	11.3	0.35
ANI–PPM	−9.80	10.9	0.39	−3.21	5.70	0.59	−0.99	6.61	0.88	0.83	4.61	0.85	−0.05	0.85	0.95	9.85	11.3	0.39
CLO–PPM	3.13	10.9	0.78	1.35	5.70	0.82	−0.16	6.61	0.98	0.92	4.61	0.84	0.03	0.85	0.97	5.55	11.3	0.63
ANI–CLO–PPM	4.97	80.9	0.95	33.6	42.1	0.44	11.4	48.9	0.82	−4.92	3.40	0.88	1.05	6.31	0.87	2.48	83.9	0.98
Statistical Values																		
RSD	3.42			1.78			2.07			1,44				0.27			8.69	
R^2^	0.99			0.99			0.99			0.99				0.99			0.95	
Adjusted R^2^	0.99			0.99			0.99			0.99				0.98			0.95	
Predicted R^2^	0.99			0.99			0.98			0.99				0.97			0.94	
PRESS	468			127			171			83.0				2.85			9893	

^1^ Total volatile fatty acids; ^2^ acetate-to-propionate ratio; ^3^ NH_3_-N (mg/100 mL).

**Table 6:** Effects of combinations of thyme oil (THY), peppermint oil (PPM), and cinnamon leaf oil (CIN) (triad 1) on rumen microbial fermentation profile assessed in vitro. Values include total volatile fatty acids (TVFAs), molar proportions, and ammonia-N (NH_3_-N) concentration.

EO Proportions (%)	Triad 1 Treatment	TVFAs (mM)	Acetate (%)	Propionate (%)	Butyrate (%)	A:P	NH_3_-N mg/100 mL
THY100	T1	40.5	45.5	18.4	29.6 *	2.38	35.6
THY67 + PPM33	T2	45.7	46.5	18.4	27.8	2.54	42.7
THY33 + PPM67	T3	44.4	45.2	19.9	27.1	2.27	43.9
PPM100	T4	48.4 *	44.7	19.4	27.4	2.30	35.7
PPM67 + CIN33	T7	45.2	47.5	20.6 *	26.6	2.31	45.0
PPM33 + CIN67	T9	46.4 *	45.6	20.9 *	25.2	2.19 *	39.9
CIN100	T10	44.5	45.0	21.4 *	26.1	2.10 *	32.7 *
THY33 + CIN67	T8	45.1	45.7	19.4	27.8 *	2.35	39.3
THY67 + CIN33	T5	43.8	44.3	19.5	28.3 *	2.28	37.8
THY33 + PPM33 + CIN33	T6	42.2	47.7	21.1 *	25.1	2.26	42.5
Control	CTR	40.4	47.2	19.8	22.9	2.40	36.4
	SEM	2.50	1.23	0.79	0.92	0.09	1.88
	*p*-Value	0.05	0.41	<0.01	<0.01	<0.01	<0.01

* Means different from control in the same column (*p* < 0.05).

**Table 7:** Possible trends based on observed results for the combination of essential oils within combination triad 1.

EO Combination	Observed Trends		
	TVFAs ^1^ (mM)	A:P Ratio ^2^	NH_3_-N ^3^ mg/100 mL
PPM 100%	48	2.30	40
CIN 80% + PPM 20% ^4^	46	2.20	36
CIN 90% + THY 10%	45	2.25	34

^1^ Total volatile fatty acids. ^2^ The acetate-to-propionate ratio. ^3^ NH_3_-N (mg/100 mL). ^4^ Selected combination.

**Table 8:** Effects of combinations of anise oil (ANI), clove leaf oil (CLO), and peppermint oil (PPM) (triad 2) on rumen microbial fermentation profile assessed in vitro. Values include total volatile fatty acids (TVFAs), molar proportions, and ammonia-N (NH_3_-N) concentration.

EO Proportions (%)	Triad 2 Treatment	TVFAs (mM)	Acetate (%)	Propionate (%)	Butyrate (%)	A:P	NH_3_-N mg/100 mL
ANI100	T1	56.2	46.9	20.8	20.9	2.26	32.7 *
ANI67 + CLO33	T2	57.2 ^+^	46.0	21.9 *	22.6 ^+^	2.11 *	37.7
ANI33 + CLO67	T3	57.8 ^+^	49.2	20.6	20.7	2.42	33.3 *
CLO100	T4	48.9	46.2	21.9 *	22.7 ^+^	2.13 *	33.7 *
CLO67 + PPM33	T7	53.1	46.1	21.3 *	23.1 ^+^	2.17 *	37.6
CLO33 + PPM67	T9	55.9	46.2	20.4	22.9 ^+^	2.27	39.2
PPM100	T10	58.8 ^+^	45.4	19.9	22.9 ^+^	2.30	40.6
ANI33 + PPM67	T8	54.8	45.0	19.9	22.6 ^+^	2.28	39.3
ANI67 + PPM33	T5	55.9	45.9	20.4	21.7	2.25	38.3
ANI33 + CLO33 + PPM33	T6	55.9	47.2	20.9	21.9	2.27	38.2
Control	CTR	55.2	50.1	20.4	19.2	2.50	38.1
	SEM	2.05	1.57	0.45	0.98	0.12	2.23
	*p*-Value	0.06	0.32	<0.01	0.10	0.04	0.05

* Means different from control in the same column (*p* < 0.05). ^+^ Means different from the control in the same column (*p* < 0.10).

**Table 9:** Possible trends based on the observed results for the combination of essential oils within combination triad 2.

EO Combination	Observed Trends		
	TVFAs ^1^ (mM)	A:P Ratio ^2^	NH_3_-N ^3^ mg/100 mL
CLO 50% + ANI 50%	58	2.30	36
PPM 90% + CLO 10%	58	2.30	40
ANI 80% + CLO 20% ^4^	57	2.10	38

^1^ Total volatile fatty acids. ^2^ The acetate-to-propionate ratio. ^3^ NH_3_-N (mg/100mL). ^4^ Selected combination.

**Table 10:** Estimated kinetic parameters of gas production derived from the application of essential oils in an in vitro experimental system using the Gompertz non-linear model. Each parameter is accompanied by its standard error (SEM) and corresponding *p*-value.

Gas Kinetic Constants	CTR	MON ^1^	T1 ^2^	T2 ^3^	SEM ^4^	*p*-Value
a, mL	0.022	0.016	0.018	0.020	0.0025	0.30
b, h^−1^	0.65	0.65	0.56	0.64	0.036	0.29
c, h	0.18 ^a^	0.18 ^a^	0.15 ^b^	0.18 ^a^	0.006	<0.01

^1^ Monensin used as positive control; ^2^ (CIN 80% + PPM 20%); ^3^ (ANI 80% + CLO 20%); ^4^ standard error of the mean; ^a^, ^b^ different superscripts in the same row indicate significant differences (*p* < 0.05).

**Table 11:** Total gas, methane production, CH_4_ to total gas ratio, and pH after 24 h of in vitro fermentation.

Item	CTR	MON ^1^	T1 ^2^	T2 ^3^	SEM ^4^	*p*-Value
Cumulative total gas (mL/24 h)	795.2 ^a^	481.0 ^b^	416.1 ^c^	481.5 ^b^	0.03	<0.01
Cumulative CH_4_ (mL/24 h)	60.4 ^a^	19.2 ^c^	17.1 ^c^	34.2 ^b^	0.01	0.01
Ratio CH_4_/total gas	0.07 ^a^	0.04 ^c^	0.04 ^c^	0.07 ^a^	0.01	0.05
pH	6.57 ^a^	6.60 ^b^	6.61 ^b^	6.64 ^c^	0.014	0.01

^1^ Monensin used as positive control; ^2^ (CIN 80% + PPM 20%); ^3^ (ANI 80% + CLO 20%); ^4^ standard error of the mean; ^a^, ^b^, ^c^ different superscripts in the same row indicate significant differences (*p* < 0.05).

**Table 12:** Comparison of observed and predicted methane (CH_4_) values for selected essential oil combinations.

EO Proportion	Treatment	Observed CH_4_ (mL/24 h)	Predicted CH_4_ ^1^ (mL/24 h)	Difference ^2^
CIN 80% + PPM 20%	T1	17.1	25.4	−8.30
ANI 80% + CLO 20%	T2	34.2	29.9	4.30

^1^ Predicted CH_4_ was calculated using the empirical equation from Foiklang et al. (2015) [54]: CH_4_ = 0.45 × %Acetate − 0.275 × %Propionate + 0.40 × %Butyrate. ^2^ The difference column represents a simple numerical subtraction (Observed – Predicted).

## Data Availability

The data presented in this study are available upon request from the corresponding author.

## Abbreviations

The following abbreviations are used in this manuscript:

GHG	Greenhouse gas
EO	Essential oil
EOB	Essential oil blend
VFA	Volatile fatty acid
TVFA	Total VFAs
A:P	Acetate-to-propionate ratio
NH_3_-N	Ammonia-N
HABP	Hyper-ammonia-producing bacteria
CH_4_	Methane
SCD	Simplex centroid design
